# Physiological, genomic, and sulfur isotopic characterization of methanol metabolism by *Desulfovibrio carbinolicus*

**DOI:** 10.1371/journal.pone.0245069

**Published:** 2021-01-14

**Authors:** Min Sub Sim, Connor T. Skennerton, Victoria J. Orphan

**Affiliations:** 1 School of Earth and Environmental Sciences, Seoul National University, Seoul, South Korea; 2 Division of Geological and Planetary Sciences, California Institute of Technology, Pasadena, California, United States of America; Universidade Nova de Lisboa, PORTUGAL

## Abstract

Methanol is often considered as a non-competitive substrate for methanogenic archaea, but an increasing number of sulfate-reducing microorganisms (SRMs) have been reported to be capable of respiring with methanol as an electron donor. A better understanding of the fate of methanol in natural or artificial anaerobic systems thus requires knowledge of the methanol dissimilation by SRMs. In this study, we describe the growth kinetics and sulfur isotope effects of *Desulfovibrio carbinolicus*, a methanol-oxidizing sulfate-reducing deltaproteobacterium, together with its genome sequence and annotation. *D*. *carbinolicus* can grow with a series of alcohols from methanol to butanol. Compared to longer-chain alcohols, however, specific growth and respiration rates decrease by several fold with methanol as an electron donor. Larger sulfur isotope fractionation accompanies slowed growth kinetics, indicating low chemical potential at terminal reductive steps of respiration. In a medium containing both ethanol and methanol, *D*. *carbinolicus* does not consume methanol even after the cessation of growth on ethanol. Among the two known methanol dissimilatory systems, the genome of *D*. *carbinolicus* contains the genes coding for alcohol dehydrogenase but lacks enzymes analogous to methanol methyltransferase. We analyzed the genomes of 52 additional species of sulfate-reducing bacteria that have been tested for methanol oxidation. There is no apparent relationship between phylogeny and methanol metabolizing capacity, but most gram-negative methanol oxidizers grow poorly, and none carry homologs for methyltransferase (mtaB). Although the amount of available data is limited, it is notable that more than half of the known gram-positive methanol oxidizers have both enzymatic systems, showing enhanced growth relative to the SRMs containing only alcohol dehydrogenase genes. Thus, physiological, genomic, and sulfur isotopic results suggest that *D*. *carbinolicus* and close relatives have the ability to metabolize methanol but likely play a limited role in methanol degradation in most natural environments.

## Introduction

Sulfate-reducing microorganisms (SRMs) utilize a great variety of organic compounds as an electron donor for energy production, being responsible for most of the terminal carbon mineralization in anoxic environments where sulfate is available [1]. Methanol is a common C_1_-compound in nature as a product of pectin and lignin degradation and also represents an inexpensive energy source for industrial bioprocesses, which is often considered as a non-competitive substrate for methanogenic archaea in sulfate-rich environments [2]. However, although less common, several species of SRMs are capable of oxidizing methanol ([3–9] and references therein). Their growth rates are usually slower than those of methanogenic microorganisms at high methanol concentrations, but SRMs have been reported to outcompete methanogens for methanol, where the environmental conditions such as methanol concentration or temperature seem more favorable for SRMs [10–13]. In methanol-fed bioreactors, stimulation of SRMs is particularly problematic because dissimilatory sulfate reduction accumulates toxic and corrosive hydrogen sulfide [14, 15]. While a role for SRMs in methanol-containing environments has been shown, knowledge about their underlying physiology remains limited.

Chemical investigation of laboratory cultures is valuable for assessing the physiology of a microorganism, but most microorganisms remain uncultured. Application of molecular and stable isotopic techniques offer a view of microbial metabolic activity *in situ*. An increasing number of genome sequences from SRMs are now available, quite a few of which have been tested for their capacity to degrade methanol. Recently, genomic and proteomic studies using a sulfate-reducing bacterium *Desulfofundulus kuznetsovii* have revealed two pathways involved in methanol degradation [9, 16], providing a solid basis to develop molecular markers for methanol oxidation coupled with sulfate reduction. Stable isotope ratios, albeit less specific, have been also used extensively as recorders of microbial activities. For example, the dominant mode of CO_2_ fixation at the given environments can be constrained by measuring the ^13^C/^12^C isotope ratio of organic compounds [17]. For the anaerobic oxidation of methanol by SRMs, carbon isotope effects have been measured [9], but no data are currently available for sulfur isotope fractionation. Depletion of heavy sulfur isotopes in sulfide relative to reactant sulfate is a well-established diagnostic for sulfate respiration, where the magnitude of sulfur isotope discrimination primarily reflects the intracellular balance between electron acceptors and donors ([18–23] and references there in). Thus, knowledge of the sulfur isotope fractionation by methanol-degrading SRMs can inform us of the efficiency of respiratory coupling between sulfate reduction and methanol oxidation.

Here we presented the full circularized genome sequence of *Desulfovibrio carbinolicus*, one of the early known sulfate-reducing bacteria capable of oxidizing methanol [3, 24] and assessed the metabolic pathways linked to this process. This work was coupled to experiments characterizing growth kinetics and sulfur isotope fractionation, focusing on the influence of alcohol metabolisms by varying the chain length of alcohols or using mixed substrates.

## Materials and methods

### Culture experiments

A series of batch culture experiments were conducted with a gram-negative, freshwater sulfate-reducing bacterium, *D*. *carbinolicus* that was originally isolated from a waste water treatment plant [3, 24]. *D*. *carbinolicus* was incubated in a chemically defined, carbonate buffered medium containing (per liter): NaHCO_3_, 5g; Na_2_SO_4_, 3g; KH_2_PO_4_, 0.2g; NaCl, 2.5g; MgCl_2_·6H_2_O, 1.3g; CaCl_2_·2H_2_O, 0.15g; KCl, 0.5g; resazurin, 1.1mg; 1 ml of trace element solution SL-10 [25]; 10 ml of vitamin solution described as a part of DSMZ medium 141 (catalogue of strains 1993; DSMZ, Braunschweig, Germany); and 1 ml of tungsten-selenium stock solution (4 mg of Na_2_WO_4_·2H_2_O and 3 mg of Na_2_SeO_3_·5H_2_O per 1 L of 12.5 mM NaOH). Sodium ascorbate (5 mM) and titanium (III) chelated by nitrilotriacetate (NTA; 60 μM) were added as a reducing agent, and C1 to C4 n-alcohols were used as an electron donor. A mixed-substrate medium consisted of 7 mM methanol and 10 mM ethanol, and the other medium contained one of the following: methanol (15 mM), ethanol (20 mM), n-propanol (20 mM), and n-butanol (20 mM). Sodium acetate (3 mM) was added as a supplementary carbon source. The medium was titrated to pH 7.2 and prepared anaerobically under 80% N_2_-20% CO_2_ gas. Culture bottles containing sterile media were inoculated with cells that had been washed three times by centrifugation and re-suspension in fresh medium without an electron donor. Unless specified otherwise, the cultures grown in their respective media were used as inocula.

All cultures were incubated at room temperature (20°C). Growth in n-alcohol cultures was monitored every 24 hours for 6 days. For cultures containing methanol, samples for chemical and sulfur isotope analyses were taken up to 3,200 hours, with the sulfate and methanol concentrations measured at 5,800 hours to confirm completion of reaction. After the cessation of regular sampling at 3,200 hours, sulfide was removed from the medium by flushing with N_2_/CO_2_ gas. Optical density at 660 nm (A660) was measured at each time point, and samples for further analyses were collected by filtering 1.5 ml of culture through a 33 mm-diameter, 0.2 μm-pore membrane (Millipore, Cork, Ireland). A 0.5 ml of filtrate was mixed with 0.1 ml of 1 M zinc acetate solution, fixing dissolved sulfide as ZnS and stored at 4°C until quantification and isotope analysis of sulfur species. The remaining aliquot of filtrate was stored at -80°C for alcohol and organic acid analyses.

Sulfide concentration was measured using a modified methylene blue assay [26], and sulfate concentration was determined *via* ion chromatography (Dionex 500, Sunnyvale, CA, USA). Concentrations of relevant organic compounds, including methanol, ethanol, acetate and formate, were measured using an Agilent 1100 HPLC (Agilient Technologies, Santa Clara, CA, USA) equipped with a UV-visible diode array detector and a refractive index detector. Samples were applied to an Aminex 87H column (Bio-Rad, Hercules, CA, USA) and separated with 8 mM sulfuric acid as an isocratic mobile phase at 0.6 ml/min. For both chromatography analyses, the concentrations were calculated by comparing the analyte peak areas to those obtained from standard solutions of varying concentrations. Analyses of sulfide and sulfate were subject to ±5% error, and the uncertainty of the HPLC results was ±10%. Specific growth rates (day^-1^) were derived from the slope of the natural logarithm of optical density versus time, and growth yields were calculated as the ratio of optical density increased per sulfate consumed (A660/mM sulfate). The specific sulfate reduction rate (mM sulfate/A660/day) was calculated from the specific growth rate and growth yield.

Sulfur isotope compositions of sulfide and sulfate were measured from the precipitated ZnS and the supernatant, respectively, using a Thermo Fischer Scientific Neptune Plus multi-collector inductively coupled plasma mass spectrometer (MC-ICP-MS) as described previously [23, 27, 28]. Sulfur isotope ratios are reported as normalized to that of the starting sulfate in the culture medium:

$$\delta^{34}S={}^{34}R_{S}/{}^{34}R_{o}-1$$ (1)

where ^34^R_s_ and ^34^R_o_ are the ^34^S/^32^S ratios of sample and starting sulfate, respectively, and the analytical uncertainty for δ^34^S was 0.2‰. Sulfur isotope enrichment factor (^34^ε) was calculated using an approximate solution to the Rayleigh distillation equation [29]:

$$1000\cdot \ln\left(1+\frac{\delta^{34}S\left(so_{4}{}^{2-}\right)}{1000}\right)=-{}^{34}\varepsilon \cdot \ln(f)$$ (2)

$$1000\cdot \ln\left(1+\frac{\delta^{34}S\left(HS^{-}\right)}{1000}\right)={}^{34}\varepsilon \cdot \left(f\cdot \ln(f)\right)/(1-f)$$ (3)

where *f* is the fraction of the remaining sulfate, δ^34^S(SO_4_^2-^) is the sulfur isotope composition of remaining sulfate, and δ^34^S(HS^-^) is isotopic composition of produced sulfide. Using linear regression analysis, values of ^34^ε were obtained from the slope of–ln*f* versus δ^34^S(SO_4_^2-^) and (*f*·ln*f*)/(1-*f*) versus δ^34^S(HS^-^). According to this definition, positive ^34^ε values represent the depletion of ^34^S in the product. All analytical errors were propagated *via* either Monte Carlo simulation (n = 5,000) or first-order Taylor series expansion [30].

### Genome sequencing and phylogenetic analysis

The genomic DNA was isolated and sheared with using a Covaris g-TUBE and size selected with AMPure XP beads. The genome of *D*. *carbinolicus* was sequenced at the Washington State University Genomics Lab, using the Pacific Biosciences RSII sequencer with P6-C4 sequencing polymerase and dye chemistry. The genome was assembled using HGAP3 into a single circular chromosome, four complete circular plasmids and a single partial plasmid fragment. Open reading frames were annotated and assigned to functional categories, using prodigal 2.6 [31]. The complete sequence of *D*. *carbinolicus* chromosome and plasmids have been deposited in GenBank under accession numbers CP026538 (*D*. *carbinolicus* chromosome), CP026539 (pDCAR1), CP026540 (pDCAR2), CP026541 (pDCAR3), CP026543 (pDCAR4) and CP026542 (pDCAR5). For the phylogenetic analysis, we used the newly genome-sequenced *D*. *carbinolicus* and 52 species of sulfate-reducing bacteria that were previously tested for methanol dissimilation and had their whole genome sequenced and deposited in the NCBI genome database. 16S rRNA gene sequences were aligned using MUSCLE with default settings, and the neighbor-joining tree was constructed using the maximum composite likelihood model and pairwise deletion in MEGA X [32]. Robustness of the phylogeny was tested using 2,000 bootstrap replicates. Since a recent proteomic study using a gram-positive sulfate-reducing bacterium *D*. *kuznetsovii* suggested that two different enzymes were involved in the dissimilation of methanol [9], a methanol methyltransferase and an alcohol dehydrogenase, their potentially homologous protein sequences were identified from the selected sulfate-reducing microorganisms using BLASTp and conserved domain searches in the NCBI database.

## Results

### Growth and sulfur isotope fractionation

**Fig 1:**
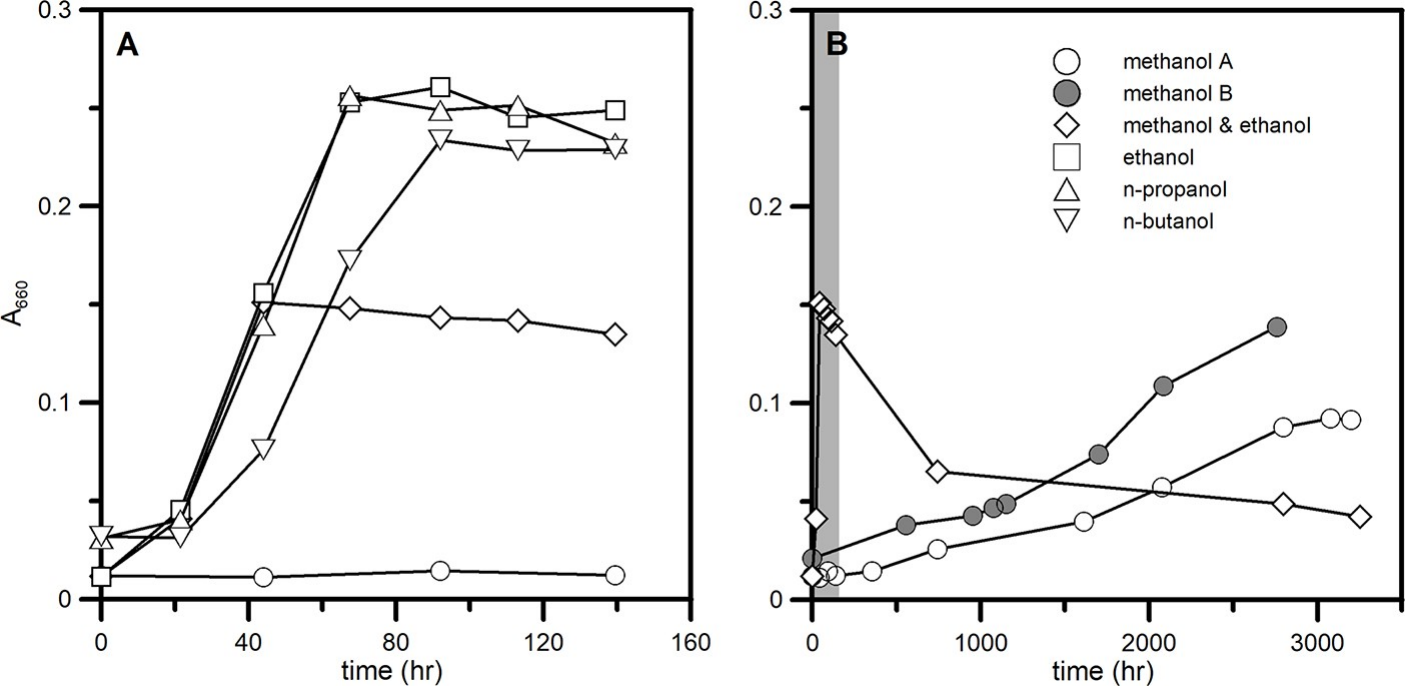
Growth of *D*. *carbinolicus* on various alcohols as electron donors in batch cultures. A shaded interval in (B) indicates the first 160 hours of the batch culture experiments (A). Methanol A represents the methanol batch culture inoculated with ethanol-grown cells, while methanol B indicates a second-generation culture inoculated with methanol A.

**Fig 2:**
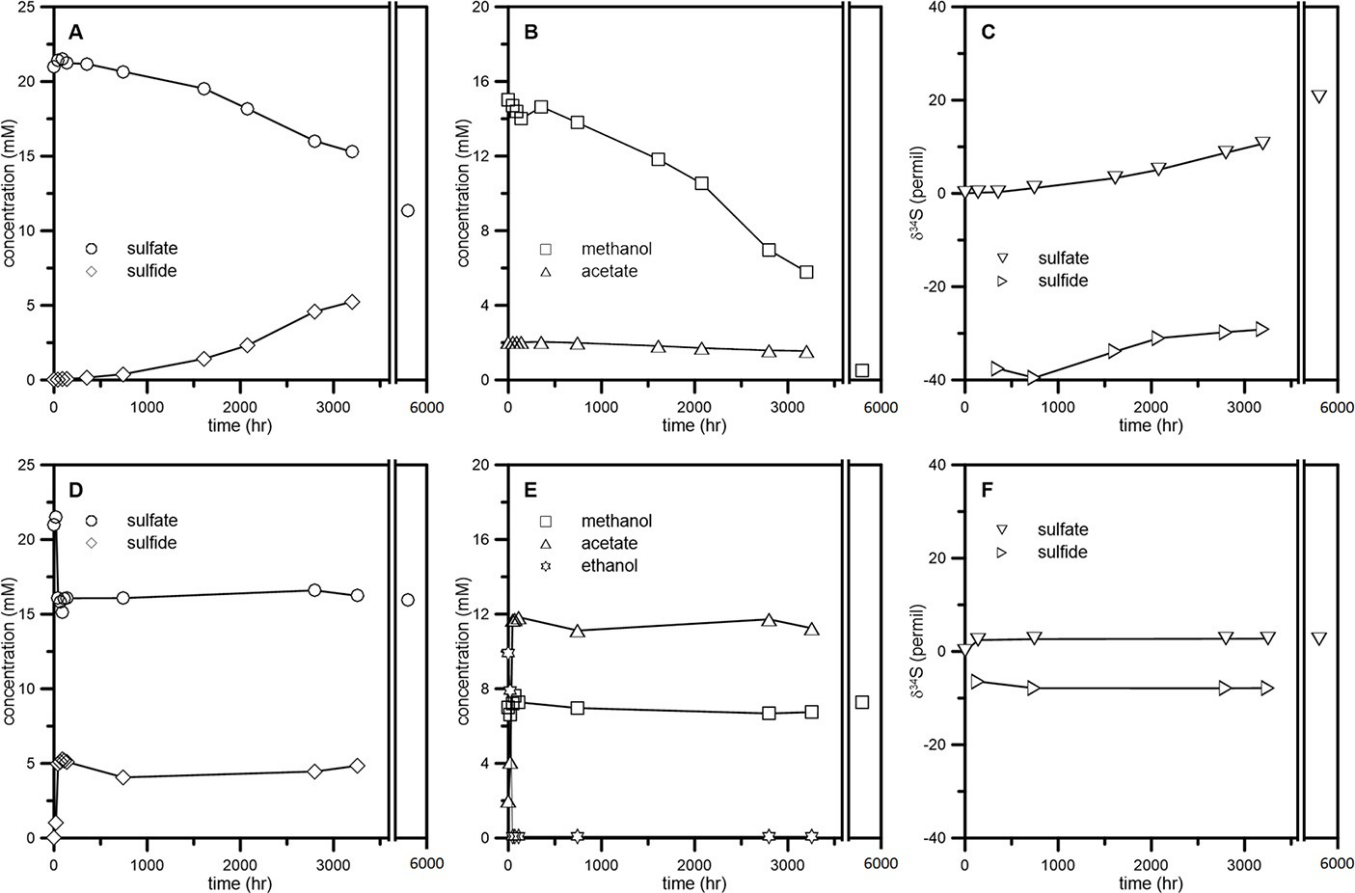
Time evolution of concentrations and sulfur isotope compositions of metabolites during the batch culture containing methanol as a sole electron donor (A-C) and ethanol and methanol as mixed substrates (D-E).

The growth of *D*. *carbinolicus* began after a short lag period (less than a day) and ended within 4 days with ethanol, n-propanol, and n-butanol as a sole electron donor (Fig 1A). The amount of sulfide produced was close to 10 mM, which agrees with the incomplete oxidation of the given primary alcohol to the corresponding carboxylic acid (Table 1). The calculated enrichment factors (^34^ε) were almost identical at 10‰ for these cultures (Table 1). No growth was observed with n-alcohols lager than n-butanol as an electron donor. When cells grown with ethanol were washed and transferred to the medium containing methanol as a sole electron donor, the changes in optical density and substrate concentrations were not apparent for the first 500 hours (Fig 1A). After a prolonged incubation up to 3,200 hours, the optical density increased to ~0.1 (Fig 1B), and the concentrations of sulfate and methanol decreased to 15 mM and 7 mM, respectively (Fig 2). The specific growth rate with methanol as an electron donor was about two orders of magnitude slower than that with ethanol, and the growth yield was halved (Table 2). No detectable formate accumulated as a metabolic product in the culture medium (< 0.2 mM). Although regular sampling was not continued after 3,200 hours, we confirmed that the concentration of methanol eventually decreased to less than below 1 mM (Fig 2). The first-generation methanol culture was sub-cultured to give a second-generation culture, and growth of both cultures was comparable when the effect of different inoculum size was accounted for (Fig 1B). During the monitored period, sulfate reduction fueled by methanol oxidation continuously enriched the remaining sulfate in the heavy isotopes (Fig 2), and the enrichment factor was estimated to be 36.1±1.0‰ (Table 2). To test for a possible diauxic growth, cells grown with methanol were transferred to the medium containing both methanol (7 mM) and ethanol (10 mM). The population grew rapidly on ethanol, while the concentration of methanol remained constant (Figs 1 and 2). During this stage, both growth kinetics and sulfur isotope fractionation were essentially similar to those of the culture with ethanol as a sole electron donor (Table 2). Once the concentration of ethanol dropped below the detection (< 0.2 mM), however, growth stopped and did not resume for more than 5,000 hours (Fig 1). Neither methanol nor sulfate was metabolized since then (Fig 2). Eliminating the metabolic waste sulfide by degassing did not change the results.

**Table 1:** Concentrations and sulfur isotope compositions of sulfur metabolites after the complete consumption of alcohols by *D*. *carbinolicus*.

initial culture medium	remaining sulfate		produced sulfide		^34^ε (‰)
	concentration (mM)	δ^34^S (‰)	concentration (mM)	δ^34^S (‰)	
21 mM sulfate, 20 mM ethanol	10.9	6.9	9.5	-6.6	9.8 ± 0.6
21 mM sulfate, 20 mM n-propanol	10.5	6.8	9.5	-6.6	9.7 ± 0.6
21 mM sulfate, 20 mM n-butanol	11.0	7.3	9.6	-6.8	10.2 ± 0.6

All concentrations and sulfur isotope compositions were determined at 140 hr (Fig 1).

**Table 2:** Comparison of growth parameters and sulfur isotope fractionations in *D*. *carbinolicus* batch cultures containing methanol and/or ethanol as electron donors.

	methanol (14 mM)	methanol (7 mM), ethanol (10 mM)	ethanol (20 mM)
growth rate (day^-1^)	0.0158±0.0002	1.39±0.04	1.12±0.02
growth yield (A660/mM sulfate)	0.012±0.001	0.023±0.006	0.027±0.004
specific sulfate reduction rate (mM sulfate/A660/day)	1.35±0.17	60.2±15.3	41.3±5.6
sulfur isotope fractionation (‰)	36.1±1.0	8.7±0.4	9.8±0.6

Calculations were done for the culture experiments shown in Figs 1 and 2, and uncertainties were propagated from the analytical errors on each measurement.

### Genomic analysis of ethanol and methanol metabolism

The genome of *D*. *carbinolicus* is comprised of 4,688,870 bp with a 64.6% GC content. Three 16S-23S-5S rRNA operons and a total of 54 tRNA genes were identified. There is only one nucleotide difference between any pair of 16S rRNA genes, resulting in the intragenomic variability below 0.05%. The genome contains 4,203 genes, 68 pseudogenes, and 4,050 coding sequences (CDS). The *D*. *carbinolicus* genome contains a complete set of genes for dissimilatory sulfate reduction and carries multiple genes encoding alcohol and aldehyde dehydrogenases involved in the oxidation of alcohol to carboxylic acid. Specifically, two genes (C3Y92_RS00555, C3Y92_RS07660) encode a 1,3-propanediol dehydrogenase (PDDH) subfamily of the type III alcohol dehydrogenase, which has been reported to be involved in methanol metabolism [9, 33, 34]. No homolog of a methanol methyltransferase was found.

*D*. *carbinolicus* is genetically very similar to *Desulfovibrio magneticus*, which is not a known methanol oxidizer but has been shown to produce magnetosomes [35]. The average nucleotide identity was 93.8% between the two genomes; however, multiple rearrangements were observed between these two genomes as well as a number of unique inserted sequences that are the basis for the phenotypic differences between them. Among these unique regions was the megnetosome biosynthesis cluster on the *D*. *magneticus* genome, while the unique regions of the *D*. *carbinolicus* genome contained three prophage and other transposable elements.

## Discussion

### Methanol oxidation in *D*. *carbinolicus*

**Fig 3:**
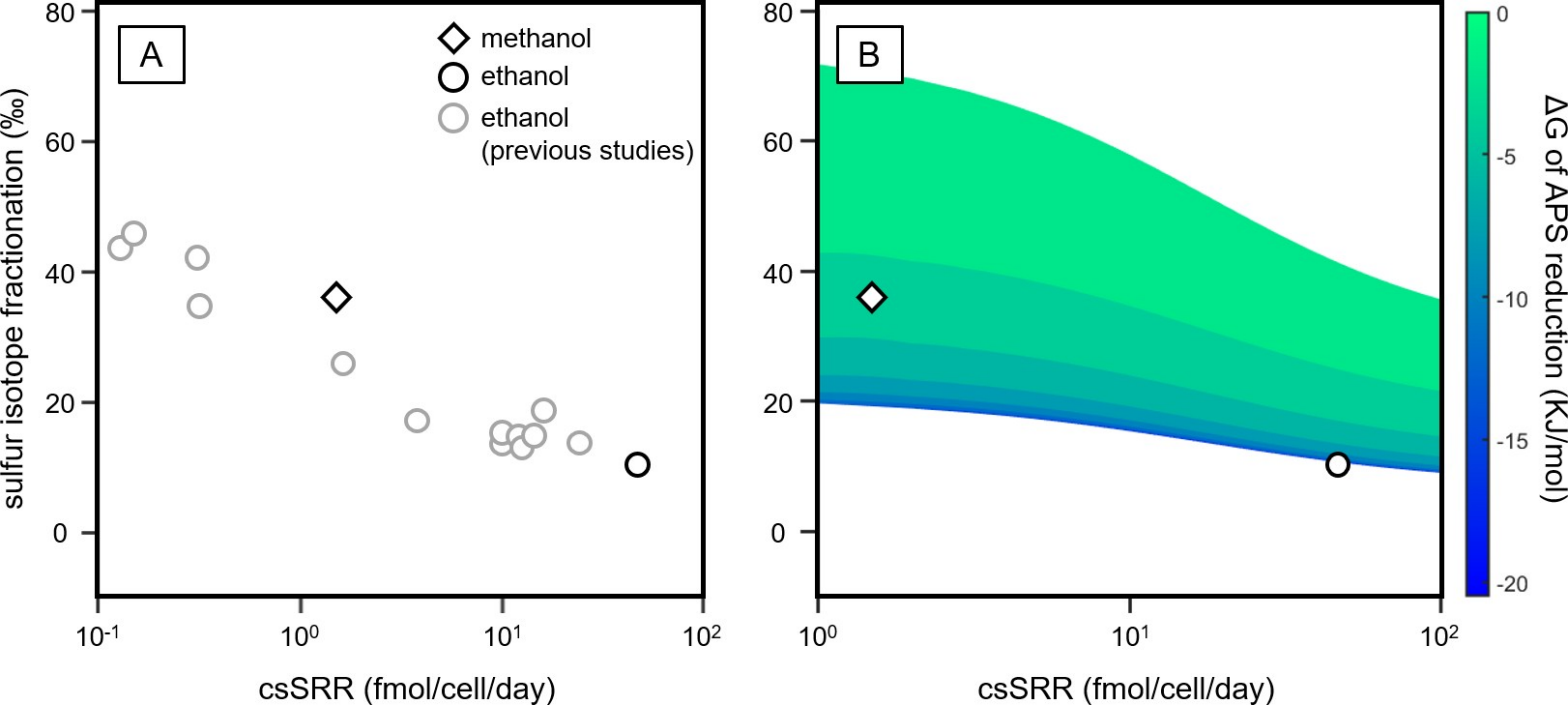
(A) Variations in specific respiration rate and sulfur isotope effect during sulfate reduction coupled to methanol or ethanol oxidation, reported in this study and previous literature [18, 48, 50, 51]. For the comparison with prior work, the respiration rate normalized by optical density is converted to the approximate cell-specific sulfate reduction rate (csSRR) according to the conversion factor for optical density to total cell volume (A660 of 1.0 as 1.49 μl/ml [52]) and the average cell volume of 1.68 μm^3^ [3]. (B) Pattern of the sulfur isotope fractionation and the free energy change (ΔG) for APS reduction, predicted based on the model originally proposed by Wing and Halevy [21] and modified by Sim et al. [45]. Sulfur isotope fractionation and free energy change are calculated as a function of both csSRR and reduction potential of the electron-donating half reaction. The former varies from 1 fmol/cell/day to 100 fmol/cell/day and the latter from -140 mV to -70 mV. All calculations are made using the constant sulfate and sulfide concentrations of 15 mM and 5 mM, respectively, which approximates when the reaction is half completed.

The nature of electron donors influences the fractionation of sulfur isotopes of the electron acceptors during microbial respiration by altering the route and rate of electron transfer to the terminal reductases [20, 36–38]. However, an increase in alcohol chain length from ethanol to n-butanol results in negligible changes in the magnitude of sulfur isotope fractionation (< 1‰), suggesting that an identical set of enzymes are likely involved in the oxidation of n-alcohols. Alcohol dehydrogenases isolated from *Desulfovibrio* species have been shown to catalyze the oxidation of n-alcohols ranging from ethanol to butanol with a lower activity toward butanol [39, 40], which might be responsible for a decrease in growth and a slight increase in ^34^ε value during the oxidation of butanol (Fig 1 and Table 1). In contrast to the similar results for n-alcohols with 2 to 4 carbon atoms, the specific rates of growth and sulfate reduction with methanol as a sole electron donor is more than an order of magnitude slower than those with other alcohols (Table 2). As described in previous studies [20, 41, 42], such slow metabolism quadruples the magnitude of sulfur isotope fractionation. Theoretically, the overall isotope effect is governed by the reversibility of each enzymatic step in the dissimilatory sulfate reduction pathway: the higher the reversibility is, the larger the sulfur isotope fractionation becomes [21]. The reversibility of each enzymatic step is dependent on the Gibb’s free energy of the reaction.

$$Reversibility=\frac{backward\;reaction\;rate}{forward\;reaction\;rate}=e^{\frac{\Delta G}{RT}}$$ (4)

where R is the gas constant, T the absolute temperature, and ΔG is the free energy change associated with the reaction. Although the standard reduction potential of methanol from CO_2_ (E_0_’ = -0.37 V) is the same order of that of ethanol from acetate (E_0_’ = -0.39 V; calculated after [43]), sulfur isotope fractionations coupled with methanol and ethanol oxidation span a wide range from less than 10‰ to over 40‰ (Fig 3A) because ΔG reflects the substrate concentrations and the kinetic properties of the involved enzymes as well as the nature of electron donors [21, 44, 45]. Sulfur isotope fractionation of 36‰ far exceeds that imparted by APS reductase and is close to the sum of the isotope effects in APS and subsequent reduction steps [45, 46], implying that the APS reduction should be reversible during the growth on methanol [45]. A model based on a flux-force relationship (Eq 4) predicts the ΔG of APS reduction in the methanol-grown culture to be about -3 kJ/mole, corresponding to the reversibility of 0.3, while this step is practically irreversible with ethanol as an electron donor (Fig 3B). Recent advances in isotope geochemistry have enabled the experimental assessment of reversibility [23, 47, 48], which is beyond the scope of this study. However, future work incorporating oxygen isotope analysis would extend our understanding of reversibility effects during sulfate respiration with methanol. A less negative free energy change suggests that methanol oxidation and subsequent electron transfer processes are sluggish as compared to ethanol. Indeed, it has been shown that alcohol dehydrogenases isolated from *D*. *carbinolicus* exhibit marginal catalytic activities for methanol oxidation [49], and here sulfur isotope data confirm previous *in vitro* results.

When presented with methanol and ethanol, *D*. *carbinolicus* first grows exclusively on ethanol, where growth kinetics and sulfur isotope fractionation are comparable to those with ethanol with a sole electron donor. Microorganisms often exhibit diauxic growth in a batch culture containing a mixture of two substrates, but *D*. *carbinolicus* consumes no methanol even after its growth on ethanol ceases. Methanol is thus consumed neither simultaneously nor sequentially, suggesting that a single enzymatic system is unlikely to be responsible for both ethanol and methanol metabolisms. In addition to alcohol dehydrogenase, a few SRMs oxidize methanol to CO_2_ *via* a methyl-transfer reaction predominantly used by methanogens and homoacetogens. This methanol methyltransferase is absent from the *D*. *carbinolicus* genome, but instead, *D*. *carbinolicus* contains multiple genes encoding alcohol dehydrogenases, two of which are homologous to the enzyme involved in methanol oxidation in other methylotrophic bacteria [9, 34]. We hypothesize these dehydrogenases are likely responsible for the oxidation of methanol by *D*. *carbinolicus*, but the regulatory element that controls the expression of methanol-oxidizing alcohol dehydrogenase currently remains unclear. An unfavorable free energy change with increasing sulfide concentration might hinder the oxidation of methanol coupled with sulfate reduction at the end of growth on ethanol, but sulfide removal by purging with N_2_/CO_2_ gas (< 20 μM) failed to resume sulfate respiration in the mixed-substrate culture. Alternatively, even low levels of residual ethanol may suppress the expression of the dehydrogenase responsible for methanol oxidation, which might be related to the energetic state of the cells after fast growth on ethanol switching to a substantially less favorable carbon substrate. Such strong suppression effects by ethanol on methanol metabolism have been described in yeast [53], although the target enzyme is not alcohol dehydrogenase but alcohol oxidase. Future studies that incorporate transcriptomic and proteomic approaches could help resolve the role of each dehydrogenase in methanol and ethanol oxidation.

Methanol is present in nature as a product of pectin and lignin degradation, but also used in industrial wastewater treatment as a cheap carbon and energy source for microbial digestion [54]. Since methanogenic, homoacetogenic, and sulfate-reducing microorganisms compete for the available methanol under anaerobic conditions, the dominant methylotrophic group may vary across different environments. However, slow methanol metabolism and its strong suppression by alternative electron donors suggest that while having a thermodynamic advantage over methanogens and homoacetogens [43], *D*. *carbinolicus* and presumably its close relatives have a limited role in methanol degradation in nature, where the complex suite of organic substrates is present. In wastewater treatment with methanol as a primary substrate, the addition of a small amount of ethanol might be a promising way to control sulfide production. This is the first report for sulfur isotope fractionation coupled with methanol oxidation, but as fractionation increases with methanol as an electron donor, sulfur isotopes may provide constraints on the role of SRMs in natural or artificial methanol-rich environments.

### Phylogenetic distribution of methanol metabolism among sulfate-reducing bacteria

**Fig 4:**
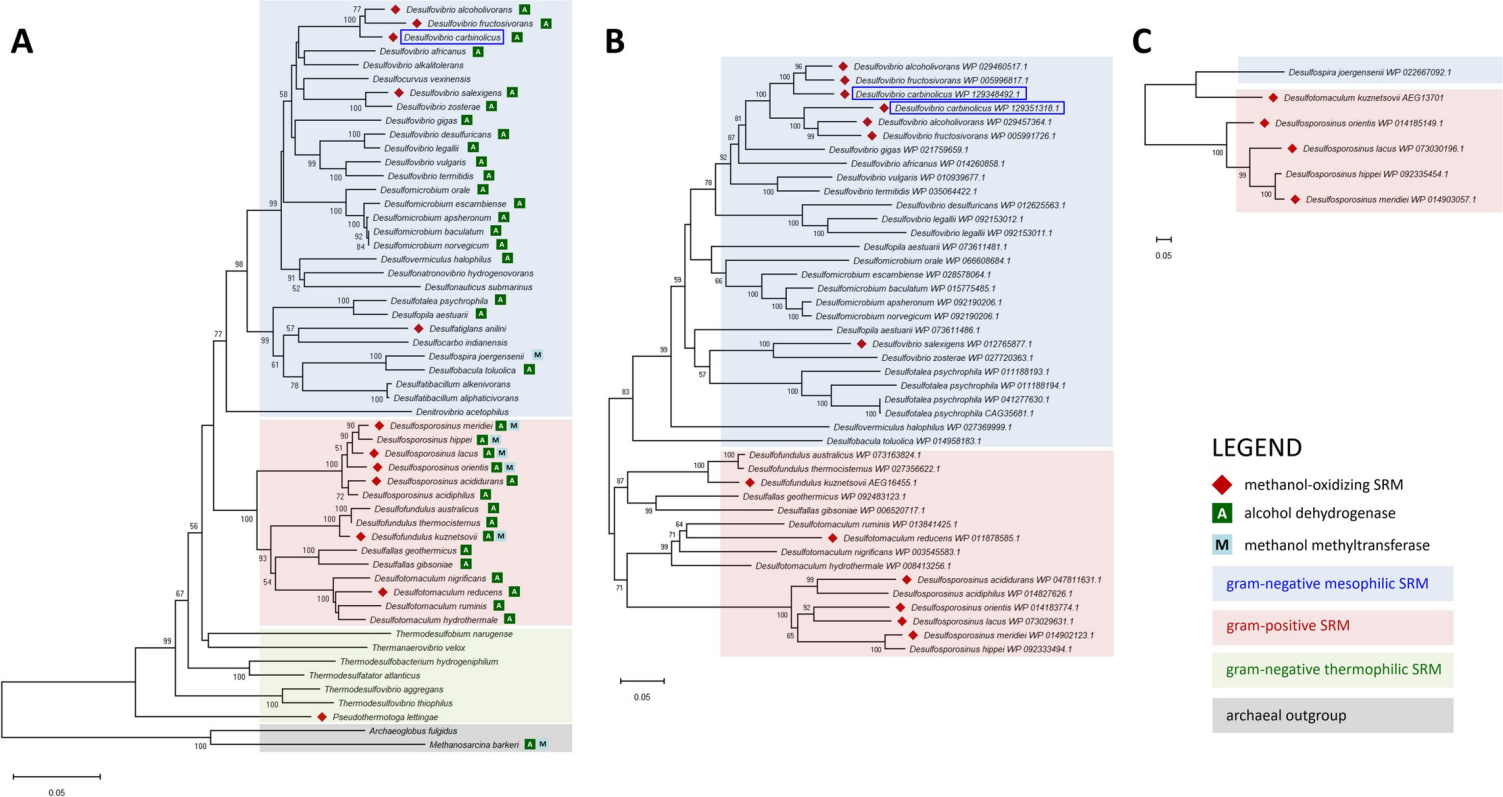
Phylogenetic tree of the selected sulfate-reducing bacteria based on 16S rRNA sequence (A) and of the genes encoding homologous proteins to methanol-oxidizing alcohol dehydrogenase (B) and methanol methyltransferase (C) of *D*. *kuznetsovii* [9]. This analysis involves 52 species of sulfate-reducing bacteria that have their genome sequenced and deposited in NCBI database and have been tested for methanol metabolism [3–8, 12, 49, 55–88]. The trees were constructed using MEGA X software with the neighbor-joining method. Numbers before each branch point represents the percentage of bootstrap resampling based on 2,000 trees. Bootstrap values below 50% are not shown. *D*. *carbinolicus* examined in this study is highlighted in a blue box.

In methylotrophic SRMs, methanol oxidation is catalyzed by either methanol methyltransferase or alcohol dehydrogenase [9, 49]. The genome of the methanol oxidizing *D*. *carbinolicus* virtually lacks a methyltransferase coding gene but contains two genes encoding the proteins homologous to the methanol-dissimilating alcohol dehydrogenase reported from methylotrophic microorganisms, including the sulfate-reducing bacterium *D*. *kuznetsovii* [9]. Similar to its closest relative, the *D*. *magneticus* genome carries two loci coding for methanol-dissimilating enzyme homologs, and the pairwise comparison reveals that more than 95% of the nucleotides are identical in the corresponding regions of the *D*. *carbinolicus* and *D*. *magneticus* genomes. Thus, *D*. *magneticus* likely has the ability to metabolize methanol, the same as *D*. *carbinolicus*, although methanol oxidation by *D*. *magneticus* has not been tested experimentally. In addition to *D*. *carbinolicus* and *D*. *magneticus*, the genomes of an increasing number of SRMs have been sequenced and annotated, over 50 species of which have been tested for their capacity to metabolize methanol in culture. Hence, their phylogenetic analyses based on the 16S rRNA gene and the genes encoding the alcohol dehydrogenase and methyltransferase may provide new insights into the evolutionary and ecological significance of methanol dissimilation by SRMs. Among the 53 species of sulfate-reducing bacteria examined, 12 are able to oxidize methanol. Based on the 16S rRNA phylogeny, methylotrophs occur in multiple clades of sulfate-reducing bacteria with non-methylotrophic sister taxa, although four species each are found in the genera of *Desulfovibrio* and *Desulfosporosinus* (Fig 4A). The genes homologous to those encoding the methanol-oxidizing alcohol dehydrogenase are identified in 35 genomes of sulfate-reducing bacteria, but only 10 of them have been shown to metabolize methanol. When narrowed down to the species containing the methyl-tranferase gene, 4 out of 6 are identified with the ability to oxidize methanol. Interestingly, two methanol-metabolizing SRMs, *Desulfatiglans anilini* and *Pseudothermotoga lettingae* [5, 7], have neither of those two, suggesting that there are likely additional, currently unknown pathways for methanol oxidation.

The enzymes homologous to the methanol-oxidizing alcohol dehydrogenase of *D*. *kuznetsovii* are distributed in most lineages of SRMs excluding the thermophilic gram-negative bacteria (Fig 4A), and their phylogeny is in broad agreement with the 16S rRNA gene tree (Fig 4B), indicating vertical transfer of the alcohol dehydrogenase genes. This cytoplasmic enzyme belongs to the PDDH subfamily of the type III NAD-dependent alcohol dehydrogenase and catalyzes the oxidation of methanol to formaldehyde in methylotrophic bacteria; however, the catalytic activity is much higher with multi-carbon alcohols compared to methanol, and it functions more efficiently in a reverse direction, reducing formaldehyde to methanol [34, 49]. Given the lack of a simple and consistent relationship between homologous genes and methanol dissimilation (Fig 4A), such kinetic properties suggest that instead of oxidizing methanol, the enzyme may have evolved either to metabolize larger alcohols or to detoxify formaldehyde [34]. Except for two gram-positive bacteria, *Desulfosporosinus acididurans* and *Desulfotomaculum reducens*, methanol oxidizing SRMs that are currently known to have alcohol dehydrogenase but no methyltransferase genes fall into the *Desulfovibrio* genus, and their methylotrophic growth is retarded compared to that with ethanol and other conventional electron donors (Table 3). As seen in *D*. *carbonolicus*, *Desulfovibrio alcoholivorans* and *Desulfovibrio salexigens* also grow extremely slowly with methanol as an electron donor and require acetate as a carbon source [6, 79]. Methanol oxidation by *Desulfovibrio fructosivorans* does not support any growth even in the presence of acetate [4]. Thus, the function of these organisms in environmental methanol cycling is likely to be limited. Although the detailed growth kinetics of *D*. *reducens* is not currently available, a gram-positive bacterium *D*. *acididurans* also showed only weak methylotrophic growth [8].

**Table 3:** Presence of methanol dissimilating enzyme homologs in SRMs capable of methanol oxidation and their growth properties.

Methanol-oxidizing SRM		PDDH	MT	Growth with methanol as an electron donor	Ref.
Gram negative	*Desulfovibrio alcoholivorans*	◯	×	+	[6]
	*Desulfovibrio carbinolicus*	◯	×	+	[3]
	*Desulfovibrio fructosivorans*	◯	×	-	[4]
	*Desulfovibrio salexigens*	◯	×	+	[79]
Gram positive	*Desulfotomaculum reducens*	◯	×	na	[60]
	*Desulfosporosinus acididurans*	◯	×	+	[8]
	*Desulfosporosinus lacus*	◯	◯	+	[88]
	*Desulfosporosinus meridiei*	◯	◯	++	[66]
	*Desulfosporosinus orientis*	◯	◯	++	[57]
	*Desulfofundulus kuznetsovii*	◯	◯	++	[68]

PDDH, 1,3-propanediol dehydrogenase subfamily of the type III alcohol dehydrogenase; MT, methyltransferase; ++, good growth comparable to that with ethanol; +, lesser growth than that with ethanol; -, methanol oxidation without growth; na, no growth kinetic information available.

Unlike alcohol dehydrogenases, the occurrence of the methyltransferase gene (mtaB) is restricted to only a few species of SRMs, and their phylogeny is not congruent with 16S rRNA phylogeny. Based on the mtaB gene sequences, the gram-positive *D*. *kuznetsovii* is positioned together with the gram-negative *Desulfospira joergensenii*, while gram-positive *Desulfosporosinus* species form a separate lineage (Fig 4C), suggesting that simple vertical inheritance is unlikely. It has been previously shown that the mtaB genes in sulfate-reducing bacteria reside in two distinct phylogenetic clades: one clade containing sequences from methanogenic archaea, while the other contains sequences from acetogenic bacteria [9]. A patchy occurrence of the mtaB gene in SRMs and a discordance of this gene tree with the species tree can be the consequence of horizontal gene transfer. Although the number of relevant examples is rather limited, all known SRMs containing the mtaB gene and capable of methanol oxidation belong to gram-positive bacteria and generally utilize methanol better than those only with alcohol dehydrogenase genes (Table 3). For example, the methylotrophic growth of *D*. *kuznetsovii*, *Desulfosporosinus orientis* and *Desulfosporosinus meridiei* was comparable to the growth with other common substrates [57, 66, 68]. However, because those gram-positive SRMs also carry the homologous genes for methanol-oxidizing alcohol dehydrogenases (Table 3), it remains to be determined whether methyltransferase pathway is advantageous for methanol oxidation as compared to dehydrogenase pathway. Given that the genes encoding the PDDH subfamily of alcohol dehydrogenases in gram-positive and -negative SRMs form two distinct lineages (Fig 4B), each might significantly differ in the activity toward methanol. In addition, the rate of methanol consumption by *D*. *kuznetsovii* was shown to be equivalent regardless of the pathway of methanol oxidation [9]. Thus, the contribution of each enzymatic system toward methanol oxidation needs to be further tested, including kinetic studies of the alcohol dehydrogenases from gram-positive and -negative SRMs, but genomic and physiological data available so far suggest that the gram-positive SRMs equipped with both enzymatic pathways should perhaps be considered first in attempts to understand the biogeochemical cycle of methanol in sulfidic environments rather than *Desulfovibrio* species.
